# Just How Big is the Schism Between the Health Sector and the Water and Sanitation Sector in Developing Countries?

**DOI:** 10.4137/EHI.S986

**Published:** 2008-08-19

**Authors:** A. A. Cronin, K. Pond

**Affiliations:** Robens Centre for Public and Environmental Health, University of Surrey, Guildford GU2 7XH, U.K

**Keywords:** water, sanitation, health, synergy

## Abstract

Water, sanitation and hygiene are all key aspects to a healthy environment but often they suffer from a lack of coherence within the sector itself and also a lack of synergy with the health sector. This is not acceptable given one quarter of all child deaths are directly attributable to water-borne disease. This lack of synergy is evident at many different layers including planning, resource allocation and donor commitment. Developing countries must, in consultation with their communities, examine their biggest health risks and allocate resources accordingly. Sustained dialogue and increased in-depth analysis are needed to find consensus and an improved synergy across these vital sectors.

## Introduction

Water, Sanitation and Hygiene (WASH) are core elements to any comprehensive environmental health plan. However, this does not necessarily mean that water and sanitation are always integral parts of health sector planning, particularly in developing country settings. Time and time again a lack of synergy between the two sectors have meant clinics, principally primary health facilities especially in developing countries, do not have adequate water and sanitation facilities. Time and time again new water and sanitation facilities are installed without proper links with the health authorities to ensure the potential public health gains are maximized. Time and time again basic health and economic analyses are not done to see where communities can be helped most per $ of Government/aid money spent and overarches the frequent mismatches between the preventative versus reactive aspects of the broader health family.

The analyses of global data sets bear out the impact of the Health/WASH schism. A staggering 25% of child mortality (under 14 years) annually are related to WASH diseases (Fewtrell et al. 2007); the figure for all mortality is over 6%. DALYs (Disability Adjusted Life Years) amount to 117 million (22% of total) for children under 14 years and 135 million DALYs (9% of total) for all age groups. Other estimates suggest that poor sanitation may be the single greatest contributing factor to the 9.7 million child deaths that occur each year (Water Aid, 2008). It is obvious that WASH service deficiencies hit children the hardest. These huge and unacceptable figures point to the conclusion that there is not a fundamental schism between the health and WASH sectors. Despite recent promising advances, there is still an urgent need to address the question, just how big is the gap between the Health sector and the WASH sector?

## The Key Disconnects

There are three aspects to this question—the gap between health and water, the gap between health and sanitation and the gap between water and sanitation/hygiene themselves. Addressing the first aspect, the gap between water and health, Bartram (2008) states that water, despite being critical to health, is typically low on the health agenda and the health system is often ill-equipped to engage with the water sector effectively. Too often professionals in the water resource management and supply in developing countries have little or no links to health counterparts and do not fully understand either the potential public health or the economic benefits of greater collaboration. Their health counterparts also work too much isolation. Increased synergy would lead to increased benefits for both. As stated by Jamison et al. (2006) ‘using full income in benefit-cost analyses of investments in health (and in health-related sectors such as education, water supply and sanitation, and targeted food transfers) would markedly increase estimates of net benefits or rates of return’.

The second gap, between health and sanitation, has been made consistently in this International Year of Sanitation. The tone was set by the Lancet stating in November 2007 that the international community’s apathy in adequately addressing sanitation suggests that the public health argument for sanitation must be made, and won, once again (The Lancet, 2007). The editorial concludes that not only the international community but also the global health community must take sanitation, which it regards as the most important public health intervention, more seriously. In a further editorial in The Lancet in March 2008, this argument is put much more forcibly ‘The shamefully weak presence of the health sector in advocating for improved access to water and sanitation is incomprehensible and completely short-sighted………the global health community is standing aside, absolving itself of responsibility, and firmly passing the buck to the water and sanitation sectors’ (The Lancet, 2008). These are indeed hard words and underline the extent of the health/WASH schism.

The WASH sector itself is not a unique solitary entity. Provision of water supply and adequate sanitation and effective hygiene promotion are conveniently lumped into the same institutional structures in Governments and Aid bodies, though there are distinct differences between each of these services. Water supply has been the traditional domain of engineers and hydrogeologists with the primary focus on the hardware components. Softer skills in water supply issues increased only when it became obvious that insufficient community involvement helped, in many instances, to sustain the cycle of build—fail—rebuild. Sanitation requires a stronger hard/soft skill combination from the onset. Hygiene promotion brings the WASH sector full cycle as it relies almost entirely on soft skills. The health sector can play a pivotal intermediate role between water, sanitation and hygiene but too often it is not in a position to take the higher ground due to inaction or inability to prioritise water and sanitation in even its own most basic facilities.

## Economic Aspects

Schieber et al. (2006) note that development assistance for health rose steadily since 1990 from about $2 billion to around $12 billion in 2004. Despite this, there has been a downward trend in the amount of assistance for water and sanitation since the middle of the 1990s. Though this trend recently appeared to be in reverse, the majority of this assistance remains concentrated around relatively few donor and recipient countries. According to Schieber et al. (2006) although official development assistance (ODA) increased to 0.33% of gross national income in 2005 after ten years of decline, this still is short of the 0.54% of GNI that is required to meet the Millennium Development Goals (MDGs) that relate to health. Scheiber et al. (2006) demonstrate that low income countries also spend the lowest percentage of GDP on health. South Asia and Sub-Saharan Africa spend the least per capita on health also while these same regions have the poorest water and sanitation coverage and face the greatest health burdens (Table 1). However, it should also be noted that development assistance for health increased to 15% by 2004; in contrast, aid to water and sanitation has fallen as a share of overall development assistance from 8% to 5% (UNDP, 2006).

Table 2 presents the assistance supplied to water and sanitation by donors from 1999 until 2006. The large increase in aid post-2004 is due to the U.S. investments in the reconstruction of Iraq and Japan’s investments in selected large projects in Asia. In contrast, health aid averaged U.S.$ 6.4 billion between 1997 and 1999 and increased to U.S.$ 8.1 billion in 2002 (WHO, 2003) and had increased to over U.S.$ 13 billion in 2005.

Most of the resources listed in Table 2 were used to finance investments in infrastructure (OECD-DAC, 2007). There is evidence that small supplies receive less attention and fewer resources than large supplies. Between 2000 and 2004 three-quarters of total bilateral support for water supply and sanitation was given by Japan, Germany, U.S.A, France and the Netherlands. More than half of the allocations were directed to Asia; 15% went to sub-Saharan Africa (OECD-DAC, 2007).

A recent WHO report (Hutton and Bartram, 2008) shows that in order to achieve the most basic target of halving the population without sustainable access to an improved water supply by 2015 developing countries need to spend U.S.$ 42 billion on new coverage. The cost of maintaining existing water supply services is estimated to total an additional U.S.$ 322 billion (Hutton and Bartram, 2008). Adding the costs related to sanitation onto this, estimated at $9.5 billion annually to meet the sanitation MDGs to 2015 (Hutton and Haller, 2004) and it becomes clear that there is a need to ensure that where limited aid is available to the water and sanitation sector that it is directed to the areas of most need. In addition, the way aid is given must be re-examined; evidence from many countries suggests that progress in sanitation, far more than in water, requires a planning frame of 10–15 years, whereas average donor cycles and national planning cycles operate over 2–3 year cycles (UNDP, 2006).

England (2007) reported that 21% of health aid was allocated to HIV in 2004, up from 8% in 2000; this figure is now approximately one quarter of health aid. However, HIV constitutes only 5% of the burden of disease in low and middle income countries as measured by DALYs lost. The world invests about 100 times more on AIDS than on clean water projects in developing countries. Two billion people do not have access to adequate sanitation, and about one billion lack clean water and in every WHO region more children are dying from diarrhoea than from HIV/AIDS (Table 3). Whilst there is no doubt that assistance for diseases like AIDS should be continued, the basic public health issues like clean water and sanitation should not be ignored at their expense.

Given the imbalance between the funding received by Health and WASH and the need to use all aid in a more effective manner, it is also fair to ask for more extensive consultation within developing communities in order to make these resource decisions. Surveys of poor communities in Benin, Viet Nam, Cambodia and Indonesia consistently found that “a clean home and village environment free of bad smells and flies” as the most important benefit identified by households, followed by convenience and health”. This suggests that villagers are acutely aware of the importance of a clean environment on many levels and may well prioritise aid in ways different to donors (UNDP, 2006).

## Ways Forward

Significant advances are being made on many fronts to bridge the schism. The International Year of Sanitation being celebrated in 2008 presents a great opportunity for the health and WASH sectors to unite in the fight for increased awareness of the health perils of inadequate sanitation. WASH professionals are increasingly using health tools and arguments for advocacy purposes, e.g. Cronin et al. *in press*. In a poll of health professionals in the British Medical Journal, sanitation was voted as the most important medical advance since 1840.

Strong economics arguments for improved WASH provision have been made which stress that not only do water and sanitation interventions contribute to improved public health in terms of helping to reduce waterborne diseases but also much other disease transmission also (Pruss-Ustun et al. 2008). Numerous studies from low income countries have proved beyond doubt that improved access to water, and the resulting increases in the quantity of water used or time used for improving hygiene are determining factors of health (Curtis and Cairncross, 2003). A return on a $1 investment in sanitation projects is over $9 (Hutton and Haller, 2004). Provision of safe water and adequate sanitation would result in gains of 320 million productive days per year in the 15- to 59-year age group and an extra 272 million school attendance days per annum; in fact over all productivity gains of U.S.$ 9.9 billion a year could be achieved (Pruss-Ustun et al. 2008). Consideration of water and sanitation as providers of economic benefits are consistent with addressing concerns about giving a higher profile to the Millennium Development Goal targets in terms of developmental funding.

Work from the 1970’s suggested that water and sanitation interventions were not cost effective (Walsh and Warren 1979) but this opinion has recently been challenged. Hutton and Haller (2004) showed through economic analysis that water and sanitation interventions are very cost effective. More recently, Hunter et al. (in prep.) have shown that investments in drinking-water provision at least in rural settings are highly cost beneficial in the developed world. As discussed above, there is already a clear rift between the health sector and the water and sanitation sector in terms of funding and so this is a good argument for looking at the advantages of providing adequate water and sanitation in a wider livelihood setting. A significant improvement in cost recovery would be realised if the wider economic benefits of the provision of water and sanitation are recognised.

In emergency response, many leading aid agencies have merged their health and WASH sections in order to maximize the public health benefits to WASH service provision. In addition, strong collaboration is evident between the Health and WASH clusters under the new emergency response mechanisms recommended by the Humanitarian Reform Initiative under the guidance of the Inter Agency Standing Committee (IASC). There is a growing realisation, in both emergencies and in sustainable development, that access to safe drinking-water and sanitation are key elements to securing livelihoods and poverty reduction.

Hence, there are many reasons for optimism but there is a long way to go. As The Lancet highlights, ‘sanitation has languished at the bottom of the international agenda for far too long and the global health community has been complicit in letting it stay there’ (The Lancet, 2008). Given that hygiene promotion is regarded as the most cost-effective public health intervention (Jamison et al. 2006), this is particularly unacceptable. Developing countries must, in consultation with their communities, examine their biggest health risks and allocate resources accordingly. Consensus at all levels is required for reducing the Health/WASH schism.

## Conclusions

Water, Sanitation and Hygiene (WASH) are core elements to any comprehensive environmental health plan. However, this does not necessarily mean that water and sanitation are always integral parts of health sector planning, particularly in developing country settings. The huge and unacceptable burden of disease associated with poor water and sanitation coverage, which impact on the poor and children the hardest, suggest that there is not a fundamental schism between the health and WASH sectors. Aid allocation trends suggest a decrease in relative importance for water and sanitation over health and education. However, more integration and collaboration across the sectors will ensure better value for money in aid spent in both sectors. Significant advances are being made on many fronts to bridge the schism. Strong economic arguments for increased synergy and the celebration of the International Year of Sanitation present joint opportunities for the health and WASH sectors to work together for common benefit.

## Figures and Tables

**Table 1.** Health expenditure in World Bank regions, 2003; from: Schieber et al. 2006 with sources World Bank, (2006); WHO, (2006).

**Regions**	**Per capita GDP ($)^1^**	**Per capita health expenditure ($)^1^**	**Total health expenditure (% of GDP)**
East Asia and Pacific	1267	64	5.1
Eastern Europe and Central Asia	2976	194	6.2
Latin America and the Caribbean	3325	225	6.9
Middle East and North Africa	2360	101	5.7
South Asia	545	24	4.4
Sub-Saharan Africa	608	38	5.2
**Income levels**			
Low income	481	22	4.6
Lower-middle income	1659	97	5.6
Upper-middle income	5596	341	6.4
High-income	30811	3466	10.7
**Global average**	**5969**	**602**	**6.0**

1 Adjusted by exchange rates.

**Table 2.** Annual average commitments to water supply and sanitation by donor type ($U.S. millions); Source: OECD-DAC, 2007.

	**1999–2000**	**2001–2002**	**2003–2004**	**2005–2006**
Bilateral	2,646	1,950	2,598	4,280
Multilateral	592	1,197	1,336	1,926
Total	3,238	3,147	3,934	6,206

**Table 3.** Distribution of causes of death among children aged <5 years (%) by WHO region and income group; source: World Health Organization, 2008.

**WHO region**	**Neonatal**	**HIV/AIDS**	**Diarrhoea**	**Measles**	**Malaria**	**Pneumonia**	**Injuries**	**Other**
African	26.2	6.8	16.6	4.3	17.5	21.1	1.9	5.6
Americas	43.7	1.4	10.1	0.1	0.4	11.6	4.9	27.9
South-East Asia	44.4	0.6	20.1	3.5	1.1	18.1	2.3	9.9
European	44.3	0.2	10.2	0.1	0.5	13.1	6.2	25.4
Eastern Mediterranean	43.4	0.4	14.6	3.0	2.9	19.0	3.2	13.5
Western Pacific	47.0	0.3	12.0	0.8	0.4	13.8	7.3	18.4
**Income group**								
Low	35.2	3.2	17.9	3.9	9.6	20.2	2.1	7.9
Lower middle	43.4	1.3	13.4	1.4	2.3	14.9	5.7	17.7
Upper middle	43.3	9.3	8.0	0.2	0.7	9.8	5.3	23.6
High	52.1	0.1	1.6	0.0	0.0	2.7	10.2	33.2
**Global**	**37.2**	**3.1**	**16.5**	**3.3**	**7.8**	**18.6**	**3.0**	**10.6**
